# The Relationship between Animal Welfare and Antimicrobial Use in Italian Dairy Farms

**DOI:** 10.3390/ani11092575

**Published:** 2021-09-02

**Authors:** Francesca Mazza, Federico Scali, Nicoletta Formenti, Claudia Romeo, Matteo Tonni, Giordano Ventura, Luigi Bertocchi, Valentina Lorenzi, Francesca Fusi, Clara Tolini, Gian Filippo Clemente, Federica Guadagno, Antonio Marco Maisano, Giovanni Santucci, Loredana Candela, Gianluca Antonio Romeo, Giovanni Loris Alborali

**Affiliations:** 1 Istituto Zooprofilattico Sperimentale della Lombardia e dell’Emilia Romagna ‘Bruno Ubertini’ (I.Z.S.L.E.R.), Via Bianchi 7/9, 25124 Brescia, Italy; francesca.mazza@izsler.it (F.M.); nicoletta_formenti@yahoo.it (N.F.); matteo.tonni@izsler.it (M.T.); giordano.ventura@izsler.it (G.V.); luigi.bertocchi@izsler.it (L.B.); valentina.lorenzi@izsler.it (V.L.); francesca.fusi@izsler.it (F.F.); clara.tolini@izsler.it (C.T.); g.clemente@izsler.it (G.F.C.); federica.guadagno@izsler.it (F.G.); antoniomarco.maisano@izsler.it (A.M.M.); giovanni.santucci@izsler.it (G.S.); giovanni.alborali@izsler.it (G.L.A.); 2 Centro di Referenza Nazionale per il Benessere Animale (CReNBA), Via Bianchi 7/9, 25124 Brescia, Italy; 3 Department of Food and Drug, Parma University, Via del Taglio 10, 43126 Parma, Italy; claudia.romeo@yahoo.it; 4 Italian Ministry of Health, Viale Giorgio Ribotta 5, 00144 Rome, Italy; l.candela@sanita.it (L.C.); ga.romeo-esterno@sanita.it (G.A.R.)

**Keywords:** dairy cows, AW, management, housing, animal-based measures, AMU, DDDAit

## Abstract

**Simple Summary:**

The purpose of this work was to investigate the relationship between animal welfare (AW) and antimicrobial use (AMU) in dairy farms due to limited knowledge in this sector. AW was assessed using a survey in 79 Italian farms housing over 15,000 cows. The average AW level was good. Nevertheless, a wide difference among farms emerged, both in terms of AW and AMU, which underlined the importance of creating a monitoring system to identify problem farms as well as virtuous farms (as positive examples). The use of antimicrobials deemed critical for human medicine by the European Medicines Agency was frequent, particularly in farms with good management. This could be due to a tendency to choose those perceived as the best antimicrobials. Nevertheless, it is possible to reduce the use of critical antimicrobials without affecting animal health and production. Farms with better management used more intramammary products for dry cows; thus, reducing AMU may require selective dry cow therapy in several herds. Two of the farms involved in the study did not use any antimicrobials, but their AW was poor, suggesting a management review including a possible reintroduction of AMU. Our study highlights the importance of implementing a tailored antimicrobials stewardship.

**Abstract:**

Information regarding the relationship between animal welfare (AW) and antimicrobial use (AMU) in dairy cows is limited. The current study aimed to investigate this relationship on Italian farms and to identify potential targets of AMU reduction. The study was performed at 79 Italian dairy farms housing over 15,000 cows during 2019. AW was scored with an on-farm protocol assessing farm management and staff training, housing systems, and animal-based measures. AMU was estimated using a defined daily dose per kg of animal biomass (DDDAit/biomass) for Italy. The median AW score was 73% (range: 56.6–86.8%). The median AMU was 4.8 DDDAit/biomass (range: 0–11.8). No relationship between the total AMU and AW was found. Management and staff training were positively associated with the use of the European Medicines Agency’s category B antimicrobials, which are critical for human medicine, and with intramammary products for dry cow therapy. In those farms, antimicrobial stewardship should aim to reduce the category B antimicrobials and selective dry cow therapy. Our results underline the importance of implementing both an integrated monitoring system (AW, AMU, etc.) and antimicrobial stewardship tailored to the specific needs of each dairy farm.

## 1. Introduction

Animal welfare (AW) has multifaceted dimensions; thus, AW should encompass an integrated approach that includes animal health, food security, and public health [1].

The relation between AW and animal health in dairy production is well-known. When farming conditions are poor, they can exacerbate health issues that lead to a reduction in AW. For instance, inadequate management and poor udder hygiene increase the risks of intramammary infection [2]. Poor housing conditions may increase the incidence of skin lesions in cows [3]. Mortality rates and the frequency of respiratory diseases were found to be higher in dairy calves housed with high stocking densities [4]. Recently, dairy cow welfare was defined as the second worst AW problem in Europe just after the welfare of sows, calves, and laying hens [5]. Contrary to the latter animal categories, there is however no specific European directive for dairy cow welfare, but several voluntary AW assessment protocols for dairy farms have been proposed either using human assessors [6,7,8] or automated systems [9,10,11]. Despite the current absence of a widely accepted standardized protocol for dairy cow welfare assessment, the proposed protocols are generally centered on the use of animal-based measures (ABMs) and non-animal-based measures [12]. Measuring animal welfare is the starting point for improving animal rearing conditions and animal wellbeing, with potential positive impacts also on animal health and antimicrobial use (AMU). It has been reported that improvements in AW and animal health can help the rationalization of AMU [13,14,15].

AMU in livestock can provide several benefits regarding animal health, disease control, and production. Nevertheless, excessive or inappropriate AMU in food-producing animals increases the risk of antimicrobial resistance, therapy failures, and, therefore, the persistence of a state of disease [16]. National and international organizations promote the collection of data on AMU. Nonetheless, an agreement for a standardized unit of measurement in animals has still to be reached [17]. Among the available metrics, those based on the concept of a defined daily dose (DDD) are used in countries such as Belgium, Denmark, and the Netherlands in their respective nationwide monitoring systems [18]. On dairy farms, antimicrobials are used to cure and prevent a variety of diseases. Mammary infections and preserving udder health during the dry period are the most common reasons for the administration of antimicrobials in cows [19,20].

In the dairy sector, various studies have been performed to estimate the level of AW [21,22] and to quantify AMU [23,24,25,26,27,28,29]. However, only limited information is available on the relationship between AMU and AW. In this study, we aimed to investigate the relationship between AW and AMU in dairy farms and to identify potential areas of improvement in terms of AMU reduction.

## 2. Materials and Methods

### 2.1. Farm Samples and Data Sources

Courtesy of cooperation with a dairy consortium, it was possible to include in the study 79 free-stall dairy farms that delivered milk for cheese production. All the selected farms had joined a voluntary AW improvement program. The study was conducted in the Lombardy region (Northern Italy) in which, according to the Italian Veterinary Database (www.vetinfo.it, accessed on 15 July 2021), more than 40% of Italian dairy cows are housed.

The state of AW was assessed through a questionnaire by two veterinarians during a farm visit. All the assessors participated in specific training provided by the Italian National Reference Centre for Animal Welfare (CReNBA). The visits were performed by a total of four evaluators (two groups of two) and all of the farms were audited between October and December 2019.

Data on AMU from the beginning of July 2019 to the end of June 2020, approximately six months before and after the visits, were collected via the National Electronic Prescription System, in which data entry is the responsibility of the farmer and the prescribing veterinarian. The average number of animals housed during the study clustered by age group (calves, heifers, and cows) was provided by the farmer during the visit.

### 2.2. Assessment of Animal Welfare

The AW level of the involved farms was assessed using a protocol included in ClassyFarm (www.classyfarm.it, accessed on 15 July 2021), a national monitoring system of the Italian Ministry of Health. This protocol was developed by the CReNBA and founded by the Italian Ministry of Health [30]. The ClassyFarm-CReNBA protocol for dairy cows in loose housing systems consists of a survey of 70 items regarding farm management and staff training (23 items), housing systems (29 items), and animal-based measures (18 items). The evaluation of each item entails either two or three options: “not acceptable” or “acceptable”; or “not acceptable”, “acceptable”, or “excellent”. The 70 items with their evaluation criteria are reported in the supplementary material of a previous study [31]. Each item has a different weight [30] and the overall AW score of the farm is calculated using a 50% contribution by farm management, staff training, and housing systems, and the remaining 50% by animal-based measures [32]. Finally, the score is expressed on a scale from 0% (lower AW) to 100% (higher AW).

### 2.3. Evaluation of Antimicrobial Use

AMU was calculated using the defined daily dose animal for Italy (DDDAit) as a standard metric. This metric was established during the development of the ClassyFarm system. The DDDAit has already been described in detail in previous studies on beef [33] and pig [34] farms. Briefly, a DDDAit of an antimicrobial medicinal product (oral or injectable) represents the amount of active ingredient, in milligrams, that should be administered per kg of live weight for each day of treatment, according to the summary of the product characteristic (SPC). Specifically, AMU was estimated as DDDAit consumed per biomass, in kg, of housed live weight (DDDAit/biomass) according to the following formula:

(1) $$\frac{\mathrm{mg}\;\mathrm{of}\;\mathrm{active}\;\mathrm{ingredient}\;\mathrm{used}÷\mathrm{DDDAit}}{\;\mathrm{cows}\times \mathrm{weight}\;\mathrm{at}\;\mathrm{risk}+\mathrm{heifers}\times \mathrm{weight}\;\mathrm{at}\;\mathrm{risk}+\mathrm{calves}\times 2\times \mathrm{weight}\;\mathrm{at}\;\mathrm{risk}\;}$$

The weights at risk for cows, heifers, and calves were also established during the development of ClassyFarm, and they were set at 600, 300, and 100 kg, respectively. As one year of AMU was considered and animals up to six months of age were classified as calves, the average number of housed calves was multiplied by two.

The daily dosage of intrauterine (IU) and intramammary products for milking (IM-LC) or dry cows (IM-DC) is usually defined in the SPCs as units per animal instead of mg per kg of live weight. In the case of IU or IM-LC products, the DDDAit was established as the number of units that should be administered per cow per day of treatment, as stated in the SPC. As this standardized approach could not be applied to IM-DC for such products, a DDDAit of 1 was considered, as described in previous studies [29,35]. Finally, the consumption of 1 DDDAit was considered to be equivalent to the treatment of 600 kg of biomass (the weight at risk of a cow) and, consequently, for those products, the DDDAit/biomass formula was corrected as follows:

(2) $$\frac{\mathrm{No}\;\mathrm{of}\;\mathrm{units}\;\mathrm{used}÷\mathrm{DDDAit}\times 600}{\;\mathrm{cows}\times \mathrm{weight}\;\mathrm{at}\;\mathrm{risk}+\mathrm{heifers}\times \mathrm{weight}\;\mathrm{at}\;\mathrm{risk}+\mathrm{calves}\times 2\times \mathrm{weight}\;\mathrm{at}\;\mathrm{risk}\;}$$

Antimicrobials administered in other forms, such as sprays, were not included in the study because assigning a DDDAit was not viable.

### 2.4. Statistical Analysis

Data collected on-farm and from existing databases (i.e., Italian Veterinary Database, National Electronic Prescription System, ClassyFarm) were managed and exported for further statistical analysis using Microsoft Excel (Microsoft Corp., Redmond, WA, USA).

Firstly, the variation in the total AMU (DDDAit/biomass) in dairy farms was analyzed through two separate linear models (LMs) with a normal error distribution. In a first model (i), we included as an explanatory variable the total AW score, whereas in a second model (ii), we considered separately all of the AW areas (farm management and staff training, housing systems, and ABMs). Secondly, we analyzed the consumption of antimicrobials belonging to the category B (“Restrict”) of the European Medicines Agency (EMA) [36] (i.e., quinolones, polymyxins, and III and IV generation cephalosporins). The variation in the use of category B antimicrobials (yes or no) was explored through two logistic regressions, including the total AW score in the first model and the three AW areas in the second model. Finally, we examined the effect of these same variables on the use of IM-DC products (DDDAit/biomass) through two LMs. In all models, the standardized value ((x-mean)/standard deviation) of farm biomass, which can be considered a proxy of farm size, was included as a covariate. The normality of residuals of the four LMs was assessed visually. All the analyses were conducted using SAS/STAT 9.4 software (SAS Institute Inc., Cary, NC, USA).

## 3. Results

Overall, the 79 farms housed 15,602 cows (median 154; range 30–805), 8698 heifers (median 91; range 0–349), and 4644 calves (median 50; range 3–194).

**Figure 1:**
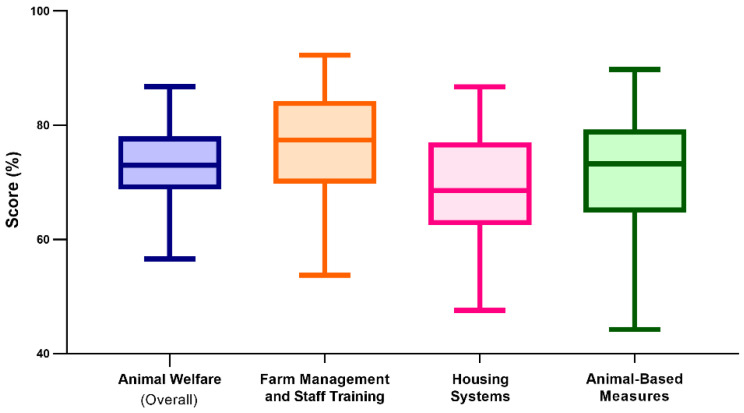
A box and whisker plot illustrating the distribution of 79 Italian dairy farm scores of the animal welfare survey.

The median AW score was 73.0%, ranging from 56.6–86.8%. The median score of farm management and staff training was 77.4% (range: 53.8–92.3%). The median score of housing systems was 68.6% (range: 47.6–86.7%). The median animal-based measures score was 73.3% (range: 44.3–89.8%). The distributions of the 79 farm scores are shown in Figure 1.

Antimicrobials were used in 77 of 79 farms (97.5%) and the median DDDAit/biomass was 4.8, ranging from zero to 11.8. Injectable products were the most used antimicrobials, representing 45.7% of the total AMU, followed by IM-DC (30.0%), IM-LC (15.5%), oral (5.7%), and IU (3.1%).

Antimicrobials included in EMA’s category B were administered in 52 of 79 farms (65.8%). The median DDDAit/biomass of category B antimicrobials was 0.1, ranging from zero to 5.2. Detailed AMU by antimicrobial class is reported in Table 1.

**Figure 2:**
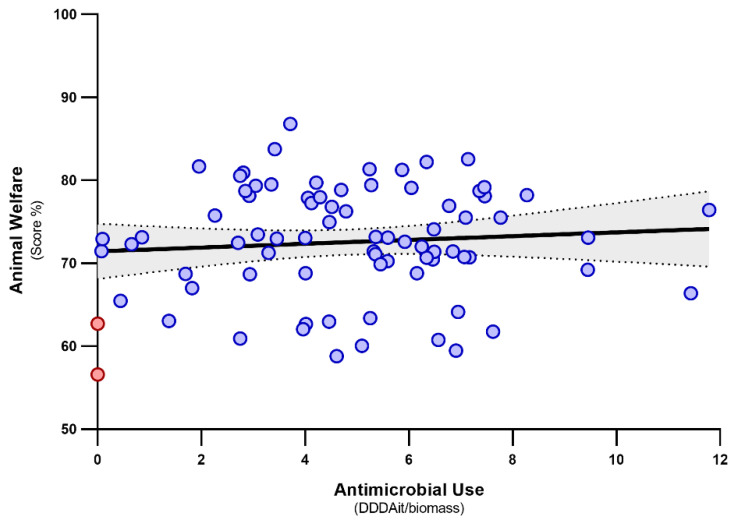
Relationship between animal welfare levels in 79 Italian dairy farms and antimicrobial use, expressed as daily dose animal for Italy per kg of biomass (DDDAit/biomass). No significant relationship was found. The grey band represents the 95% confidence interval. The red dots represent farms that did not use any antimicrobials.

**Figure 3:**
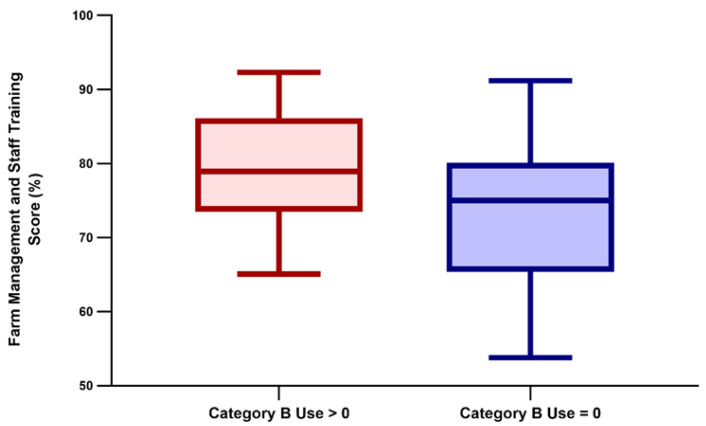
A box and whisker plot illustrating the distribution of farm management and staff training scores in farms that either used (*n* = 52) or did not use (*n* = 27) category B antimicrobials. Significant differences were found between these two groups.

The total AMU on the examined farms was neither related to the overall AW score (Figure 2) nor to any of its components, and farm biomass had no effect on AMU either (all *p* > 0.05). Conversely, the probability of using category B antimicrobials, not related to the total AW score, varied significantly with the scoring of farm management and staff training (*p* = 0.016); the full results of the logistic regression models are reported in Table 2. In detail, a 10% increase in the score of farm management and staff training led to an over two-fold increase in the probability of using category B antimicrobials (odds ratio: 2.51; 95% confidence interval: 1.19–5.30). The distribution of farm management and staff training scores in farms that either used or did not use category B antimicrobials are shown in Figure 3.

Finally, the use of IM-DC increased with farm biomass and was positively related to the score of farm management and staff training (*p* = 0.017 and *p* = 0.011, respectively). In this case, a 0.1 increase in the score led, on average, to an AMU increase of 0.25 ± 0.09 DDDA/biomass. Detailed results of the linear model are reported in Table 3.

## 4. Discussion

The AW scores of the 79 investigated farms are similar to those described in previous Italian studies conducted with the same protocol on a smaller sample of farms in Lombardy [32] and Sardinia [37]. The median overall AW score of the investigated farms was over 70%, which can be considered suitable for the evaluation protocol used in this study [31]. Nevertheless, wide differences among farms were found, which suggests that there is still considerable room for improvement in AW.

AMU varied largely among the investigated farms too, similar to that reported in previous studies [23,25,29]. However, comparing these results in quantitative terms with previous studies was not feasible because of the lack of a common standard. The majority of the available studies focused on AMU in adult cows with less information available on the use in calves [23]. In this study, it was not possible to differentiate AMU by age group, as this information was usually not present in the data source. Intramammary (IM-LC and IMD-DC) and injectable products represented more than 90% of AMU and were almost equally consumed (45.5% vs. 45.7%). This result is not necessarily in contrast with previous studies, which reported the intramammary route as the most prevalent [23,27,29,38] but were based on AMU in adult cattle only. Overall, the high consumption of intramammary antimicrobials found in this study confirms the relevance of mastitis treatment and prevention in terms of AMU.

Category B antimicrobials are pivotal for treating human infections and their use in animals should be limited [36]. Although the median use of these antimicrobials was not high (0.1 DDDAit/biomass), they were administered in more than 65% of the investigated farms with AMU and, in the highest consumer, up to more than 5 DDDAit/biomass. Almost 70% of the category B antimicrobials were third and fourth-generation cephalosporins, whose extensive use is widely documented in dairy farming [23,28,29,38]. The use of polymyxins was low (0.3% of the total AMU), which is consistent with the steady decrease in colistin sales in Italy, which fell by more than 90% between 2015 and 2018 [39].

No significant relationships emerged between the total AMU and AW scores, which might be due to the limited sample size. In addition, it may be possible that fewer antimicrobials were administered in several of the less-welfare-conscious farms because poor attention was paid to the animals and their diseases. Conversely, a group of farms with both high levels of AW and low AMU were found and this may represent a positive driver to reduce AMU for other farmers, in particular in a scenario such as the one described in this study where all the farms deliver milk to the same dairy consortium and are part of an AW improvement plan. These results also highlight the importance of setting up a monitoring system to identify both virtuous and problematic farms. The positive relationship between the score of farm management and staff training and the use of category B antimicrobials could be due to a tendency of more management-conscious farms to choose what are perceived to be the best antimicrobials. This finding underlines a relevant area to focus on for an improvement in terms of antimicrobial stewardship, particularly considering that those antimicrobials do not seem to offer any advantages in treating most mastitis cases [40], and it should be possible to reduce or even remove them without impairing production and animal health [41]. Considering the positive relationship between IM-DC use and farm management and staff training scores, reducing AMU in farms with good management may be challenging. In several farms, for instance, it may be necessary to implement selective dry cow therapy. Nevertheless, when such a strategy is implemented properly (e.g., using teat sealants in healthy cows), it should not lead to any disadvantages compared to a blanket therapy, both in terms of animal health and milk production [42].

Two of the farms included in this study no longer used antimicrobials. Nevertheless, both scored low on AW, with one being the worst; a finding, which, although limited, may be a warning of the risks, in terms of AW, of eliminating the use of antimicrobials entirely.

Although the study included over 15,000 cows, the number of involved farms was not high (79) and the sample was not randomly selected. Hence, the results may not be representative of Italian dairy farming in terms of AW or AMU.

Measures used to assess welfare may range in precision. For example, variability can be high among the observers and among the days of observation. Environment-based measures often tend to be more reliable than animal-based measures. Any protocol requiring human assessors increases the risk of intra and interobserver measurement variability compared to an automated system [43,44]. To reduce these risks, in the present study, all welfare assessments were performed by two auditors working together who had previously participated in specific training provided by the CReNBA.

Another important limitation of this study was the lack of information on AMU by age group because such data were not often available from the investigated sources. Nevertheless, it is reasonable to assume that AMU was not evenly distributed in the three age groups but was concentrated in calves because their young age increases their susceptibility to infections, and in cows, mainly due to mastitis. Thus, future studies investigating AMU in Italian dairy farms should also consider the consumption per age group.

## 5. Conclusions

In this study, we generally found good levels of AW but with sizeable differences between farms. AMU also varied widely among farms and the use of category B antimicrobials, particularly third and fourth-generation cephalosporins, was frequent. Although no direct relationship emerged between overall AMU and AW, a group of farms that excelled in both was found, which may serve as a positive example for other farmers. Finding these examples, as well as problematic farms, is possible only when a monitoring system is in place. Farms that paid particular attention to management tended to consume a larger number of IM-DC products and to administer category B antimicrobials more frequently. Our results underline the importance of implementing both an integrated monitoring system (AW, AMU, etc.) and an antimicrobial stewardship tailored to the specific needs of each dairy farm (e.g., a reduction in category B antimicrobials and selective dry cow therapy).

## Figures and Tables

**Table 1:** Distribution of the overall antimicrobial use by class in dairy farms (*n* = 79). The percentage of farms that used a given class at least once during the study is also reported (use > 0) as well as medians and ranges.

Class	Total of AMU ^1^(%)	Farms with Use > 0 (%)	Median DDDAit/Biomass (Range)
Aminopenicillins	14.1	84.8	0.495 (0–2.333)
Cephalosporins (1st–2nd Gen.)	13.6	73.4	0.299 (0–3.481)
Aminoglycosides ^2^	13.4	93.7	0.509 (0–1.951)
Penicillin (antistaphylococcal) ^3^	12.6	75.9	0.268 (0–2.895)
Penicillins	11.5	74.7	0.272 (0–2.455)
Rifamycins	7.5	60.8	0.062 (0–3.753)
Sulphonamides ^4^	7.0	70.9	0.136 (0–1.99)
Tetracyclines	4.6	68.4	0.053 (0–1.441)
Cephalosporins (3rd–4th Gen.)	4.4	51.9	0.011 (0–5.066)
Macrolides	4.3	53.2	0.037 (0–1.646)
Lincosamide	3.9	69.6	0.106 (0–1.018)
Fluoroquinolones	1.8	50.6	0.005 (0–1.07)
Amphenicols	1.0	60.8	0.011 (0–0.3)
Polymyxins	0.3	6.3	0 (0–0.693)

^1^ Antimicrobial use; ^2^ including aminocyclitols; ^3^ beta-lactamase-resistant penicillins (e.g., cloxacillin and dicloxacillin); ^4^ including trimethoprim.

**Table 2:** Logistic regression models exploring variation in the probability of using B-category antimicrobials in dairy farms (*n* = 79). Significant *p*-values are highlighted in bold.

Model	Explanatory Variables	Parameter Estimate ± Standard Error	Type III ANOVA	*p*-Value
i	Animal welfare score	4.23 ± 3.68	Χ^2^1 = 1.32	0.25
	Farm biomass	0.28 ± 0.27	Χ^2^1 = 1.10	0.30
ii	Farm management and staff training	9.22 ± 3.74	Χ^2^1 = 6.08	**0.016**
	Housing systems	−2.96 ± 3.35	Χ^2^1 = 0.78	0.38
	Animal-based measures	−0.53 ± 2.54	Χ^2^1 = 0.04	0.83
	Standardized biomass	0.07 ± 0.28	Χ^2^1 = 0.06	0.81

**Table 3:** Linear models exploring variation among dairy farms (*n* = 79) in intramammary antimicrobials usage (DDDA/biomass) in dry cows. Significant *p*-values are highlighted in bold.

Model	Explanatory Variables	Parameter Estimate ± Standard Error	Type III ANOVA	*p*-Value
i	Animal welfare score	1.44 ± 1.06	F_1, 76_ = 1.84	0.18
	Farm biomass	0.18 ± 0.07	F_1, 76_ = 5.92	**0.017**
ii	Farm management and staff training	2.46 ± 0.95	F_1, 74_=6.71	**0.011**
	Housing systems	0.07 ± 0.93	F_1, 74_=0.01	0.94
	Animal-based measures	−0.47 ± 0.70	F_1, 74_=0.46	0.50
	Standardized biomass	0.11 ± 0.07	F_1, 74_=2.35	0.13

## Data Availability

All the datasets considered in this study contain sensitive information and cannot be made publicly accessible but they are available with the anonymization of the participants from the corresponding author upon reasonable request.

## References

[1] De Passille A.M., Rushen J. (2005). Food safety and environmental issues in animal welfare. Rev. Sci. Tech..

[2] Pankey J.W. (1989). Premilking udder hygiene. J. Dairy Sci..

[3] Bernhard J.K., Vidondo B., Achermann R.L., Rediger R., Muller K.E., Steiner A. (2020). Carpal, tarsal, and stifle skin lesion prevalence and potential risk factors in Swiss dairy cows kept in tie stalls: A cross-sectional study. PLoS ONE.

[4] Losinger W.C., Heinrichs A.J. (1997). Management practices associated with high mortality among preweaned dairy heifers. J. Dairy Res..

[5] Broom D.M. (2017). Animal Welfare in the European Union.

[6] Whay H.R., Main D.C.J., Green L.E., Webster A.J.F. (2003). Assessment of the welfare of dairy cattle using animal-based measurements: Direct observations and investigation of farm records. Vet. Rec..

[7] Welfare Quality (2009). Welfare Quality. Welfare Quality Assessment Protocol for Cattle. Welfare Quality Assessment Protocol for Cattle (without Veal Calves).

[8] van Eerdenburg F.J.C.M., Di Giacinto A.M., Hulsen J., Snel B., Stegeman J.A. (2021). A New, Practical Animal Welfare Assessment for Dairy Farmers. Animals.

[9] Steinmetz M., von Soosten D., Hummel J., Meyer U., Danicke S. (2020). Validation of the RumiWatch Converter V0.7.4.5 classification accuracy for the automatic monitoring of behavioural characteristics in dairy cows. Arch. Anim. Nutr..

[10] Ruuska S., Kajava S., Mughal M., Zehner N., Mononen J. (2016). Validation of a pressure sensor-based system for measuring eating, rumination and drinking behaviour of dairy cattle. Appl. Anim. Behav. Sci..

[11] Borchers M.R., Chang Y.M., Tsai I.C., Wadsworth B.A., Bewley J.M. (2016). A validation of technologies monitoring dairy cow feeding, ruminating, and lying behaviors. J. Dairy Sci..

[12] EFSA Panel on Animal Health and Welfare (AHAW) (2012). Scientific Opinion on the use of animal-based measures to assess welfare of dairy cows. EFSA J..

[13] Diana A., Lorenzi V., Penasa M., Magni E., Alborali G.L., Bertocchi L., De Marchi M. (2020). Effect of welfare standards and biosecurity practices on antimicrobial use in beef cattle. Sci. Rep..

[14] Isomura R., Matsuda M., Sugiura K. (2018). An epidemiological analysis of the level of biosecurity and animal welfare on pig farms in Japan and their effect on the use of veterinary antimicrobials. J. Vet. Med. Sci..

[15] Stygar A.H., Chantziaras I., Toppari I., Maes D., Niemi J.K. (2020). High biosecurity and welfare standards in fattening pig farms are associated with reduced antimicrobial use. Animal.

[16] Chantziaras I., Boyen F., Callens B., Dewulf J. (2014). Correlation between veterinary antimicrobial use and antimicrobial resistance in food-producing animals: A report on seven countries. J. Antimicrob. Chemother..

[17] Collineau L., Belloc C., Stärk K.D.C., Hemonic A., Postma M., Dewulf J., Chauvin C. (2017). Guidance on the Selection of Appropriate Indicators for Quantification of Antimicrobial Usage in Humans and Animals. Zoonoses Public Health.

[18] Sanders P., Vanderhaeghen W., Fertner M., Fuchs K., Obritzhauser W., Agunos A., Carson C., Hog B.B., Andersen V.D., Chauvin C. (2020). Monitoring of Farm-Level Antimicrobial Use to Guide Stewardship: Overview of Existing Systems and Analysis of Key Components and Processes. Front. Vet. Sci..

[19] Ruegg P.L. (2017). A 100-Year Review: Mastitis detection, management, and prevention. J. Dairy Sci..

[20] Stevens M., Piepers S., De Vliegher S. (2019). The effect of mastitis management input and implementation of mastitis management on udder health, milk quality, and antimicrobial consumption in dairy herds. J. Dairy Sci..

[21] von Keyserlingk M.A.G., Weary D.M. (2017). A 100-Year Review: Animal welfare in the Journal of Dairy Science-The first 100 years. J. Dairy Sci..

[22] Blanco-Penedo I., Ouweltjes W., Ofner-Schrock E., Brugemann K., Emanuelson U. (2020). Symposium review: Animal welfare in free-walk systems in Europe. J. Dairy Sci..

[23] de Campos J.L., Kates A., Steinberger A., Sethi A., Suen G., Shutske J., Safdar N., Goldberg T., Ruegg P.L. (2021). Quantification of antimicrobial usage in adult cows and preweaned calves on 40 large Wisconsin dairy farms using dose-based and mass-based metrics. J. Dairy Sci..

[24] Ferroni L., Lovito C., Scoccia E., Dalmonte G., Sargenti M., Pezzotti G., Maresca C., Forte C., Magistrali C.F. (2020). Antibiotic Consumption on Dairy and Beef Cattle Farms of Central Italy Based on Paper Registers. Antibiotics.

[25] Firth C.L., Käsbohrer A., Schleicher C., Fuchs K., Egger-Danner C., Mayerhofer M., Schobesberger H., Kofer J., Obritzhauser W. (2017). Antimicrobial consumption on Austrian dairy farms: An observational study of udder disease treatments based on veterinary medication records. PeerJ.

[26] Hyde R.M., Remnant J.G., Bradley A.J., Breen J.E., Hudson C.D., Davies P.L., Clarke T., Critchell Y., Hylands M., Linton E. (2017). Quantitative analysis of antimicrobial use on British dairy farms. Vet. Rec..

[27] Kuipers A., Koops W.J., Wemmenhove H. (2016). Antibiotic use in dairy herds in the Netherlands from 2005 to 2012. J. Dairy Sci..

[28] Redding L.E., Bender J., Baker L. (2019). Quantification of antibiotic use on dairy farms in Pennsylvania. J. Dairy Sci..

[29] Stevens M., Piepers S., Supre K., Dewulf J., De Vliegher S. (2016). Quantification of antimicrobial consumption in adult cattle on dairy herds in Flanders, Belgium, and associations with udder health, milk quality, and production performance. J. Dairy Sci..

[30] Bertocchi L., Fusi F., Angelucci A., Bolzoni L., Pongolini S., Strano R.M., Ginestreti J., Riuzzi G., Moroni P., Lorenzi V. (2018). Characterization of hazards, welfare promoters and animal-based measures for the welfare assessment of dairy cows: Elicitation of expert opinion. Prev. Vet. Med..

[31] Ginestreti J., Strano R.M., Lorenzi V., Fusi F., Angelucci A., Ferrara G., Galletti G., Bergagna S., Bolzoni G., Zanardi G. (2020). Bulk tank milk quality data is unlikely to give useful information about dairy cow welfare at herd level. J. Dairy Res..

[32] Ginestreti J., Lorenzi V., Fusi F., Ferrara G., Scali F., Alborali G.L., Bolzoni L., Bertocchi L. (2020). Antimicrobial usage, animal welfare and biosecurity in 16 dairy farms in Lombardy. Large Anim. Rev..

[33] Diana A., Santinello M., Penasa M., Scali F., Magni E., Alborali G.L., Bertocchi L., De Marchi M. (2020). Use of antimicrobials in beef cattle: An observational study in the north of Italy. Prev. Vet. Med..

[34] Scali F., Santucci G., Maisano A.M., Giudici F., Guadagno F., Tonni M., Amicabile A., Formenti N., Giacomini E., Lazzaro M. (2020). The Use of Antimicrobials in Italian Heavy Pig Fattening Farms. Antibiotics.

[35] Scherpenzeel C.G.M., den Uijl I.E.M., van Schaik G., Riekerink R.G.M.O., Lam T.J.G.M. (2014). Evaluation of the use of dry cow antibiotics in low somatic count cows. J. Dairy Sci..

[36] European Medicines Agency (EMA) Answer to the Request from the European Commission for Updating the Scientific Advice on the Impact on Public Health and Animal Health of the Use of Antibiotics in Animals—Categorisation of Antimicrobials (EMA/CVMP/CHMP/682198/2017). https://www.ema.europa.eu/en/documents/other/answer-request-european-commission-updating-scientific-advice-impact-public-health-animal-health-use_en.pdf.

[37] Loi F., Pilo G., Franzoni G., Re R., Fusi F., Bertocchi L., Santucci U., Lorenzi V., Rolesu S., Nicolussi P. (2021). Welfare Assessment: Correspondence Analysis of Welfare Score and Hematological and Biochemical Profiles of Dairy Cows in Sardinia, Italy. Animals.

[38] Larde H., Dufour S., Archambault M., Masse J., Roy J.P., Francoz D. (2021). An observational cohort study on antimicrobial usage on dairy farms in Quebec, Canada. J. Dairy Sci..

[39] European Surveillance of Veterinary Antimicrobial Consumption (ESVAC) Sales of Veterinary Antimicrobial Agents in 31 European Countries in 2018 (EMA/24309/2020). https://www.ema.europa.eu/en/documents/report/sales-veterinary-antimicrobial-agents-31-european-countries-2018-trends-2010-2018-tenth-esvac-report_en.pdf.

[40] Nobrega D.B., Naqvi S.A., Dufour S., Deardon R., Kastelic J.P., De Buck J., Barkema H.W. (2020). Critically important antimicrobials are generally not needed to treat nonsevere clinical mastitis in lactating dairy cows: Results from a network meta-analysis. J. Dairy Sci..

[41] Turner A., Tisdall D., Barrett D.C., Wood S., Dowsey A., Reyher K.K. (2018). Ceasing the use of the highest priority critically important antimicrobials does not adversely affect production, health or welfare parameters in dairy cows. Vet. Rec..

[42] Kabera F., Roy J.P., Afifi M., Godden S., Stryhn H., Sanchez J., Dufour S. (2021). Comparing Blanket vs. Selective Dry Cow Treatment Approaches for Elimination and Prevention of Intramammary Infections During the Dry Period: A Systematic Review and Meta-Analysis. Front. Vet. Sci.

[43] Molina F.M., Marin C.C.P., Moreno L.M., Buendia E.I.A., Marin D.C.P. (2020). Welfare Quality(R)for dairy cows: Towards a sensor-based assessment. J. Dairy Res..

[44] Stygar A.H., Gomez Y., Berteselli G.V., Dalla Costa E., Canali E., Niemi J.K., Llonch P., Pastell M. (2021). A Systematic Review on Commercially Available and Validated Sensor Technologies for Welfare Assessment of Dairy Cattle. Front. Vet. Sci..

